# Dynamic parameters of fluid responsiveness in the operating room: An analysis of intraoperative ventilation framework conditions

**DOI:** 10.1007/s00101-024-01428-y

**Published:** 2024-06-28

**Authors:** M. Prütz, A. Bozkurt, B. Löser, S. A. Haas, D. Tschopp, P. Rieder, S. Trachsel, G. Vorderwülbecke, M. Menk, F. Balzer, S. Treskatsch, D. A. Reuter, A. Zitzmann

**Affiliations:** 1 Department of Anaesthesiology, Intensive Care Medicine and Pain Therapy, University Medical Centre Rostock, Schillingallee 35, 18057 Rostock, Germany; 2 The Hirslanden Clinical Trial Unit, Hirslanden AG, Glattpark, Switzerland; 3 Institute for Anaesthetics and Intensive Care, Klinik Beau-Site, Hirslanden AG, Bern, Switzerland; 4 Department of Anaesthesiology and Surgical Intensive Care, University Medical Centre Schleswig-Holstein, Campus Kiel, Kiel, Germany; 5 Department of Anaesthesiology and Critical Care Medicine, University Hospital Carl Gustav Carus and Carl Gustav Carus Faculty of Medicine, TU Dresden, Dresden, Germany; 6 Department of Anaesthesiology and Intensive Care Medicine, Charité—Universitätsmedizin Berlin, Berlin, Germany; 7 Institute of Medical Informatics, Charité—Universitätsmedizin Berlin, Berlin, Germany

**Keywords:** Intraoperative monitoring, Fluid therapy, Pulse pressure, General anesthesia, Retrospective study, Intraoperative Überwachung, Flüssigkeitstherapie, Pulsdruck, Allgemeinanästhesie, Retrospektive Analyse

## Abstract

**Background:**

Reliable assessment of fluid responsiveness with pulse pressure variation (PPV) depends on certain ventilation-related preconditions; however, some of these requirements are in contrast with recommendations for protective ventilation.

**Objective:**

The aim of this study was to evaluate the applicability of PPV in patients undergoing non-cardiac surgery by retrospectively analyzing intraoperative ventilation data.

**Material and methods:**

Intraoperative ventilation data from three large medical centers in Germany and Switzerland from January to December 2018 were extracted from electronic patient records and pseudonymized; 10,334 complete data sets were analyzed with respect to the ventilation parameters set as well as demographic and medical data.

**Results:**

In 6.3% of the 3398 included anesthesia records, patients were ventilated with mean tidal volumes (mTV) > 8 ml/kg predicted body weight (PBW). These would qualify for PPV-based hemodynamic assessment, but the majority were ventilated with lower mTVs. In patients who underwent abdominal surgery (75.5% of analyzed cases), mTVs > 8 ml/kg PBW were used in 5.5% of cases, which did not differ between laparoscopic (44.9%) and open (55.1%) approaches. Other obstacles to the use of PPV, such as elevated positive end-expiratory pressure (PEEP) or increased respiratory rate, were also identified. Of all the cases 6.0% were ventilated with a mTV of > 8 ml/kg PBW and a PEEP of 5–10 cmH_2_O and 0.3% were ventilated with a mTV > 8 ml/kg PBW and a PEEP of > 10 cmH_2_O.

**Conclusion:**

The data suggest that only few patients meet the currently defined TV (of > 8 ml/kg PBW) for assessment of fluid responsiveness using PPV during surgery.

## Abstract (de)

**Hintergrund:**

Eine zuverlässige Beurteilung der intraoperativen Flüssigkeitsreagibilität unter Verwendung der Pulsdruckvariation (PPV) hängt von bestimmten beatmungsbezogenen Voraussetzungen ab. Diese stehen häufig im Widerspruch zu den Empfehlungen einer lungenprotektiven Beatmung.

**Ziel der Arbeit:**

Ziel dieser Studie war die retrospektive Analyse medizinischer und intraoperativer Beatmungsdaten im Hinblick auf die Anwendbarkeit der PPV bei Patienten während nicht-kardiochirurgischer Operationen.

**Material und Methoden:**

Retrospektive Daten von 10.334 Patienten aus dem Zeitraum von Januar bis Dezember 2018 aus drei großen medizinischen Zentren in Deutschland und der Schweiz wurden anhand elektronischer Patientenakten pseudonymisiert analysiert. Die Auswertung erfolgte hinsichtlich verschiedener Beatmungsparameter, demografischer und medizinischer Daten.

**Ergebnisse:**

Von den 3398 eingeschlossenen Anästhesiedatensätzen wurden die Patienten in 6,3 % der Fälle mit einem mittleren Tidalvolumen (mTV) > 8 ml/kg idealem Körpergewicht (IKG) beatmet. Diese würden sich für ein PPV-basiertes Flüssigkeitsmanagement qualifizieren, aber die Mehrheit der Patienten wurde mit niedrigeren mTV beatmet. Bei Patienten, die sich einer abdominellen Operation unterzogen (75,5 % der analysierten Fälle), wurde bei 5,5 % der Patienten ein mTV > 8 ml/kg IKG verwendet, wobei es keinen Unterschied zwischen laparoskopischem (44,9 %) und offenem (55,1 %) Zugangsweg gab. Auch andere Bedingungen für die Verwendung von PPV, beispielsweise der positive end-exspiratorische Druck (PEEP), die Atemfrequenz oder der Herzrhythmus wurden untersucht. 6,0 % aller analysierten Fälle wurden mit einem mTV > 8 ml/kg IKG und einem PEEP von 5–10 cmH_2_O beatmet und 0,3 % mit einem mTV > 8 ml/kg IKG und einem PEEP > 10 cmH_2_O.

**Diskussion:**

Die Ergebnisse deuten darauf hin, dass nur wenige Patienten die derzeit erforderlichen Beatmungsparameter erfüllen, um intraoperativ PPV zur Beurteilung der Flüssigkeitsreagibilität anzuwenden. Die Limitationen der Anwendung von PPV sollten im klinischen Alltag Berücksichtigung finden. Eine vorsichtige Interpretation, insbesondere in Bezug auf etablierte Schwellenwerte, kann dazu beitragen, die Genauigkeit und Effizienz von PPV-gesteuerten intraoperativen Flüssigkeitstherapien zu verbessern.

## Brief introduction

Dynamic preload parameters, e.g., pulse pressure variation (PPV), are employed for individualized fluid management guidance. Specific requirements must be met for reliable application of these parameters, especially ventilation strategies; however, particularly tidal volume (TV) seems to be at odds with current recommendations for lung protective ventilation.

This study analysed intraoperative ventilation parameters to determine if they are suitable for the application of dynamic parameters of fluid responsiveness and compatible with lung protective ventilation strategies.

## Introduction and background

The use of pulse pressure variation (PPV) is commonly recommended to predict intraoperative fluid responsiveness [8, 16]. To reliably assess fluid responsiveness by this parameter using accepted thresholds, the presence of sinus rhythm during controlled mechanical ventilation with a tidal volume (TV) of > 8 ml/kg predicted body weight (PBW) is essential [12, 14, 15, 21, 27]. Other more debated requirements include a low positive end-expiratory pressure (PEEP) ≤ 5 cmH_2_O [11, 21], closed chest [26] and normal intra-abdominal pressure [6, 18], which conflicts with the capnoperitoneum in minimally invasive laparoscopic surgery.

The application of TV of > 8 ml/kg PBW challenges daily clinical practice because it conflicts with the standards of a lung protective ventilation strategy. Adopted from ventilation in acute respiratory distress syndrome (ARDS), low TVs (6–8 ml/kg PBW) proved to be beneficial in other populations and have become the preferred intraoperative approach [9, 20]. Common thresholds for dynamic preload parameters, such as PPV, have been investigated for higher TVs, raising the question of their applicability under current ventilation strategies [12, 15].

This study aimed to evaluate intraoperative ventilation parameters with respect to the feasibility of using PPV to guide fluid therapy in three large tertiary care centers in Germany and Switzerland. The primary endpoint of this study was to determine the number of patients undergoing surgery who met mTV/kg PBW criteria at certain PEEP levels for a reliable assessment of PPV by specialty. Secondary endpoints included further ventilation data, such as respiratory rate, inspiratory pressure, end-tidal carbon dioxide (etCO_2_), respiratory system compliance, and cardiac rhythm.

## Study design and investigation methods

### Study design

This study was conducted as a multicenter retrospective study and three large tertiary care centers participated in data collection: Charité Universitätsmedizin Berlin (Germany), Hirslanden Hospital Network (Switzerland), and University Medical Center Rostock (Germany).

### Data collection

This study was approved by the Ethics Committee of the Medical Faculty of the University of Rostock, Germany (chairperson Prof Dr A. Büttner; file number A 2017-0220). It was consented by the respective local committees: Charité Universitätsmedizin Berlin, Germany (chairperson Prof Dr R. Stahlmann, file number EA4/239/19) and Cantonal Ethics Committee for Research Bern, Switzerland (chairperson Prof Dr C. Seiler, BASEC-Nr. Req-2018-00883).

We requested anesthesia records of all adult patients (18 years and older) who underwent surgery with general anesthesia between January and December 2018 and received a total of 63,685 pseudonymized anesthesia records. These were checked for completeness regarding medical history, demographic and ventilation data (minimum requirements in Table 1). A total of 10,334 datasets met these inclusion criteria and were reassessed for exclusion criteria (Table 1): (1) duration of surgery < 120 min, (2) etCO_2_ < 3.44 kPa or > 6.84 kPa and (3) cardiac surgery. Only those datasets that met all the specified criteria were included in the analysis.

**Table 1:** Inclusion and exclusion criteria

Minimum requirements: given values on			Exclusion criteria
Ventilation data	Demographic data	Medical data	
tidal or minute volume respiratory rate inspiratory pressure PEEP etCO_2_	age height weight sex	surgical specialty duration of surgery	duration of surgery < 120 min etCO_2_ < 3.44 kPa or > 6.84 kPa cardiac surgery

Minimum requirements for ventilation, demographic and medical data were set for inclusion in the analysis. Only records that met all the specified inclusion and exclusion criteria were included. *PEEP* positive end-expiratory pressure, *etCO2* end-tidal partial pressure of carbon dioxide

Ventilation data were recorded and extracted from the patient data management system (PDMS) at predetermined 5‑min intervals and averaged over time. Corresponding parameters, such as mean tidal volume per predicted body weight (mVT/kg PBW), were calculated from the averaged data. The PBW was calculated using the Devine formula: for men PBW = 50 kg + 0.91 ×(height (cm) − 152.4 cm) and for women PBW = 45.5 kg + 0.91 × (height (cm) − 152.4 cm). Subsequently, the mean PEEP was classified into three groups (< 5 cmH_2_O; 5–10 cmH_2_O; > 10 cmH_2_O) and mTV was grouped into mTV < 6 ml/kg PBW, mTV = 6–8 ml/kg PBW, and mTV > 8 ml/kg PBW.

In addition to demographic parameters, medical and surgical data, including diagnosis, name and duration of the procedure, and American Society of Anesthesiologists (ASA) physical status were analyzed.

Surgery was divided into categories by specialty. Abdominal procedures included urological, gynecological, and general surgical procedures. A distinction was made between laparoscopic and non-laparoscopic procedures. The second category included trauma/orthopedic as well as neurosurgery cases and the third category included thoracic cases.

### Statistical analysis

All hospitals used a customized version of COPRA (COPRA System GmbH, Berlin, Germany) as PDMS and data were extracted into Excel sheets (Microsoft Excel, Microsoft Corporation, Redmond, WA, USA). Statistical Product and Services Solutions (SPSS) software (IBM SPSS Inc, Armonk, NY, USA) was used for statistical analysis. Demographic and medical data were expressed as means ± SD together with the range. Where applicable, frequency is stated.

## Results

**Fig. 1:**
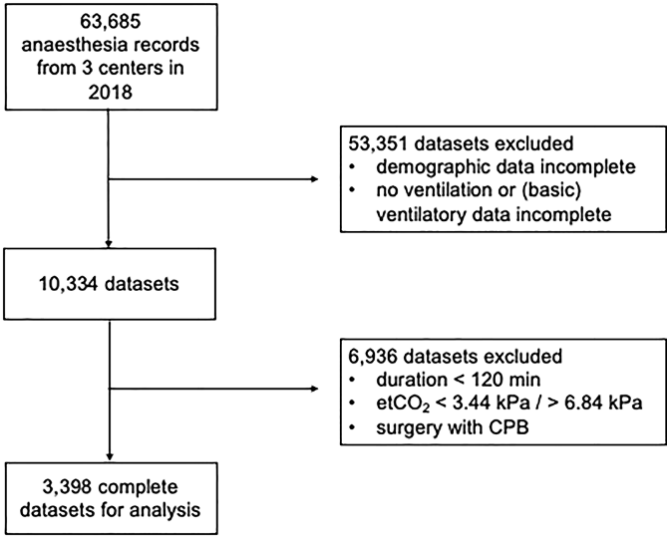
Exclusion flow chart: flow chart of the exclusions prior to analysis. *etCO*_*2*_ end-tidal carbon dioxide, *CPB* cardiopulmonary bypass

A total of 63,685 anesthesia records were received from the participating centers. After applying the exclusion criteria described above, 3398 records remained for further analysis (see Fig. 1), with 7.1% of the data evaluated from the Hirslanden Hospital Network, 63.3% from Charité Universitätsmedizin Berlin and 29.4% from the University Medical Centre Rostock.

Demographic and medical data of the 3398 datasets analyzed are presented in Table 2.

**Table 2:** Demographic and medical data

	*n*	Mean (± SD)	Minimum	Maximum
Age (years)	3398	59.8 (± 14.68)	18	96
Sex (m/f %)	3398	53.6%/46.4%	–	–
Height (m)	3398	1.71 (± 0.09)	1.53	2.07
Weight (kg)	3398	82.0 (± 20.9)	40.0	246.0
BMI (kg/m^2^)	3398	27.74 (± 6.41)	14.87	75.11
ASA physical status (1/2/3/4/5/missing %)	3241	7.7%/42.0%/40.1%/5.3%/0.2%/4.6%	–	–

Data are presented in mean ± standard deviation (*SD*) and minimum/maximum where applicable or percentage (%)

*m* male, *f* female, *BMI* body mass index (kg/m^2^), *ASA* American Society of Anesthesiologists

Excluding procedures lasting less than 120 min, the mean operating time was 228.5min (± 109.6min). Abdominal procedures accounted for 75.5% of the data analyzed, composed of 33.9% using a laparoscopic approach and 41.6% a non-laparoscopic approach. Further, 18.6% were assigned to trauma/orthopedic surgery and neurosurgery and 2.6% procedures to thoracic surgery. Of the interventions, 3.3% could not be clearly categorized to a specialty or approach.

Heart rhythm was documented in 25% (*n* = 850) of the evaluated data and classified as sinus rhythm in 93%.

Ventilation data for the overall collective are shown in Table 3; in Table 4 they are categorized by surgical specialty.

**Table 3:** Ventilation data

	*n*	Mean (± SD)	Minimum	Maximum
Mean tidal volume (ml/kg PBW)	3398	6.71 (± 0.84)	2.43	11.33
Mean PEEP (cmH_2_O)	3398	6.53 (± 1.45)	5	15.62
Mean inspiratory pressure (cmH_2_O)	3398	19.5 (± 3.9)	11.6	35.8
Mean etCO_2_ (kPa)	3398	4.61 (± 0.34)	3.45	6.72
Mean compliance (ml/cmH_2_O)	3398	40.4 (± 10.7)	9.1	85.6

Data are presented in mean ± standard deviation (*SD*) and minimum/maximum

*etCO*_*2*_ end-tidal partial pressure of carbon dioxide, *PEEP* positive end-expiratory pressure, *PBW* predicted body weight

**Table 4:** Ventilation data by the surgical field

	Abdominal surgery		Thoracic surgery	Trauma/orthopedic surgery/neurosurgery	Uncategorized
	Laparoscopic approach	Open approach			
*n*	1152	1414	89	632	111
Mean tidal volume (ml/kg PBW)	6.65 (± 0.83)	6.72 (± 0.79)	5.79 (± 0.9)	6.96 (± 0.83)	6.55 (± 0.79)
Mean PEEP (cmH_2_O)	6.9 (± 1.45)	6.41 (± 1.41)	6.74 (± 1.73)	6.06 (± 1.33)	6.52 (± 1.22)
Mean inspiratory pressure (cmH_2_O)	21.24 (± 3.82)	18.35 (± 3.66)	21.07 (± 3.81)	18.54 (± 3.47)	19.45 (± 3.85)
Mean etCO_2_ (kPa)	4.74 (± 0.34)	4.54 (± 0.31)	4.51 (± 0.4)	4.55 (± 0.33)	4.56 (± 0.3)
Mean Compliance (ml/cmH_2_O)	36.14 (± 9.8)	43.35 (± 10.65)	32.99 (± 9.85)	42.74 (± 9.52)	38.86 (± 10.77)

Data are presented in mean ± standard deviation (*SD*)

*etCO*_*2*_ end-tidal partial pressure of carbon dioxide, *PEEP* positive end-expiratory pressure, *PBW* predicted body weight

In 97.4% of all included cases, a mean PEEP in the range of 5–10 cmH_2_O was applied, while in 2.6%, mean PEEP greater than 10 cmH_2_O was used. Of the higher mean PEEP levels of > 10 cmH_2_O, 88.6% were recorded during abdominal surgery with equal distribution between laparoscopic and non-laparoscopic approaches. During thoracic surgery, only mean PEEP values between 5–10 cmH_2_O were set. The mean respiratory rate recorded was 12.8 (± 1.7) breaths/min, with a maximum of 26.1 breaths/min.

Of all included cases 75% were ventilated with a mTV of 6–8 ml/kg PBW, while a mVT greater than 8 ml/kg PBW was applied in 6.3%. Lower mTVs of less than 6 ml/kg PBW were used in 18.6%. In abdominal surgery, 5.5% were ventilated with mTVs greater than 8 ml/kg PBW, which did not differ between laparoscopic and non-laparoscopic approaches (contributing 44.9% and 55.1%, respectively to abdominal surgery). None of the recorded cases of thoracic surgery were ventilated with a mTV greater than 8 ml/kg PBW.

The data show that 6.0% of all cases included in the study were ventilated with a mTV of > 8 ml/kg PBW and a PEEP of 5–10 cmH_2_O, while 0.3% were ventilated with a mTV > 8 ml/kg PBW and a PEEP of > 10 cmH_2_O.

## Discussion

Dynamic preload parameters, such as PPV, are beneficial for goal-directed fluid management during intraoperative procedures [8]. The rationale of this retrospective analysis was to determine whether intraoperative ventilation meets the criteria for using dynamic preload parameters in daily clinical practice.

In the analyzed cases the magnitude of the TV appears to be the main point of contestation. Within the population studied, only a small proportion met the target TV of greater than 8 ml/kg PBW, which continues to be regarded as mandatory for the correct assessment of fluid responsiveness with the accepted cut-off values [12, 15], albeit meta-analysis suggesting a “fair performance” of PPV during lower TVs [1]. Lower TVs were preferred for intraoperative ventilation. This is most likely due to following a lung protective ventilation strategy [10]. Lower TVs prompt less variation in intrathoracic pressure, which may then not be high enough to trigger sufficiently large preload changes to deflect PPV, despite fluid responsiveness above common thresholds [12, 15].

The effect of elevated PEEP on PPV has been debated with conflicting results. While some studies reported good predictive quality at PEEP levels > 5 cmH_2_O [22], other authors argued that cardiac filling and subsequently PPV are affected by higher PEEP levels [11, 21]; however, as there is no standardized value for PEEP for the accurate assessment of fluid responsiveness using PPV at its common thresholds, it was necessary to categorize the data into different PEEP groups and analyze different levels of PEEP in a subgroup analysis. In the majority of our cases, a mean PEEP of 5–10 cmH_2_O was applied, which is recommended to prevent atelectasis [4, 10]. Raising PEEP reduces venous return, leading to a decrease in left ventricular preload and, thus, higher PPV values. Nevertheless, these high values should be interpreted with care and should not result in uncritical intraoperative fluid therapy, as these changes resolve after extubation.

Furthermore, De Backer et al. found that high respiratory rates limit the informative value of preload parameters [3]. In short respiratory cycles, notably at respiratory rates of 30–40/min, preload changes were attenuated, leading to reduced PPV. In this study, the mean respiratory rate was markedly below these respiratory rates, suggesting that respiratory rates did not limit the assessment of fluid responsiveness in an intraoperative setting.

One-lung ventilation (OLV) is a controversially discussed restriction of dynamic preload indicators, as it might affect pressure transfer from the ventilator to intrathoracic vessels [24]. In the recording of the data, no distinction was made between one-lung ventilation and ventilation of both lungs. The OLV is a standard anesthesia technique to facilitate thoracic surgery. For these reasons, data of patients undergoing thoracic surgery cannot be analyzed collectively with those of other surgical disciplines; thus, data with possible OLV were evaluated separately. In this group no patient had a mTV greater than 8 ml/kg PBW. This is not astonishing, as the use of low TVs during OLV is recommended to prevent acute lung injury, as these volumes are applied to a single ventilated lung [4, 7, 23]. There are data to support the applicability of PPV for predicting fluid responsiveness during OLV [13]; however, it is not common practice.

For one third of all cases analyzed, a laparoscopic approach was chosen for intra-abdominal surgery. Pneumoperitoneum during laparoscopic approach increases intra-abdominal pressure, which might affect the applicability of PPV. Therefore, laparoscopic abdominal surgeries were analyzed separately. Studies suggest that this parameter remains indicative when threshold values are increased [5]. Research is required to define thresholds for the use of preload parameters under elevated abdominal pressures.

Sinus rhythm is another absolute requirement [17] to use PPV as a guide for intraoperative fluid therapy. Arrhythmia leads to variations in stroke volume and pulse pressure independent of heart-lung interactions. In our routine care anesthesia records, cardiac rhythm was only documented in one quarter of the evaluated cases, as this had to be performed manually by the anesthesiologist. While the vast majority of cases where cardiac rhythm was documented showed sinus rhythm, it is not possible to extrapolate this to the whole study group.

The analyzed data indicate that lung protective ventilation strategies are applied in the majority of patients undergoing surgery. A different approach is a tidal volume challenge, where a transient increase in TV to at least 8 ml/kg PBW for 1min is initiated prior to determining PPV [15, 19]. In order to evaluate fluid responsiveness by PPV in as large a patient population as possible, current thresholds need to be reconsidered in different intraoperative settings.

### Limitations

We gathered a dataset with more than 63,000 anesthesia records, each containing at least 6 variables per 5‑min interval. During the first review, we discovered that the parameters were heterogeneous. They were recorded in different units, not only from hospital to hospital, but also within the individual data set, especially for TV (ml or l) and etCO_2_ (kPa or mmHg). The major issue was that the respective unit was not attached to the value provided by the ventilator. Thus, each dataset had to be analyzed individually. Each value was checked for plausibility and appropriate units were assigned accordingly. Conversion of units was particularly difficult with those values that were outside the normal range of both units, as a distinction had to be made between a particularly low value in one unit and a very high value in the other. Secondly, it was not possible to only collect data from procedures carried out with the patient under general anesthesia with controlled mechanical ventilation as requested. The chart received values from every anesthesia machine that was turned on after the electronic record had been linked to the respective workstation, irrespective of the type of anesthesia (monitored anesthesia care, regional, neuraxial or general anesthesia).

As the primary task was to identify patients with controlled mechanical ventilation as a prerequisite for the accurate use of dynamic variables for fluid responsiveness, a series of filters were applied. As procedures characteristically performed with bag-mask or laryngeal-mask ventilation are seldomly employed in clinical practice for procedures exceeding 2h [2, 25], any procedures lasting less than 120 min were excluded. Furthermore, by excluding all procedures with a duration < 120 min, it should be ensured that the evaluation is based on a sufficient number of valid measurements. Moreover, interventions with an averaged etCO_2_ of less than 3.44 kPa (80% of the lower standard value of etCO_2_) were excluded, as etCO_2_ is not accurately measured during monitored anesthesia care or bag-mask ventilation due to high leakage in the ventilation circuit, often resulting in very low etCO_2_ values. To account for the abovementioned unit conversion issue, we excluded datasets with etCO_2_ values that exceeded the upper standard value by more than 20% (equivalent to 6.84 kPa) together with the values that came below the lower standard value by more than 20% (3.44 kPa).

In addition, the intraoperative use of cardiopulmonary bypass, common in cardiac surgery, confounds the averaged ventilation data. Depending on the anesthesiologist, either no ventilation was applied (with the ventilator completely turned off or in manual/spontaneous mode), or the ventilator was set to HLM mode with minimal ventilation during extracorporeal circulation. As both forms decrease the averaged etCO_2_, minute volume, and respiratory rate, cardiac surgery was excluded.

A considerable number of records were excluded from the analysis due to incomplete datasets. This meticulous selection process aimed to prioritize datasets that met strict criteria for completeness and reliability, while maintaining the overall integrity of the research, taking into account that patients meeting the applicability criteria for PPV might have been excluded. Ventilation data were extracted and averaged by the PDMS. Data validation was required by the attending anesthesiologist prior to data release; however, the inclusion of implausible data in the calculation cannot be excluded. By averaging the values, no statement can be made as to whether the parameters were not temporarily fulfilled at another time during surgery. Furthermore, the data from Charitè—Universitätsmedizin Berlin accounted for more than two thirds of the total data. Despite the absence of any significant discrepancies between the centers, the individual hospitals exhibited different influences on the final analysis.

The predominance of abdominal surgery in the datasets can be attributed to the prevalence of general surgical procedures which are recorded by the PDMS in different hospital settings. Furthermore, the increasing use of regional anesthesia and neuraxial techniques in trauma surgery resulted in the exclusion of considerable trauma surgery data. It is important to note that our study does not reflect accurate frequency of abdominal surgical surgery in clinical practice.

## Conclusion

- Our data suggest that only a minority of all patients undergoing anesthesia currently meet recommended tidal volumes for assessing fluid responsiveness by pulse pressure variation (PPV). This implies that ventilation requirements are at odds with current lung protective ventilation strategies.
- The use of PPV as a guiding factor for intraoperative fluid therapy requires critical revaluation. Individualized adjustments to ventilation settings, such as a “tidal volume challenge”, may be necessary to ensure reliable control of fluid therapy during surgical procedures.
- Although meta-analysis suggests a fair accuracy of PPV when lower tidal volumes are applied, it is important to recognize the limitations of this method in everyday clinical practice.
- By interpreting PPV with respect to commonly accepted thresholds in a cautious manner, clinicians can improve the accuracy and effectiveness of PPV-guided fluid management strategies.
